# Supplementary material for: Direct observation of hot-electron-enhanced thermoelectric effects in silicon nanodevices
Source: Nat Commun. 2023 Jun 22;14:3731. doi: 10.1038/s41467-023-39489-z (PMC10287675; doi:10.1038/s41467-023-39489-z)
Supplement: Supplementary file 1 — Supplementary Information [file 41467_2023_39489_MOESM1_ESM.pdf]

Supplementary Information

Direct Observation of Hot-Electron-Enhanced Thermoelectric Effects in Silicon Nanodevices

Huanyi Xue[1,†], Ruijie Qian[1,2,†], Weikang Lu[1,3,†], Xue Gong[1], Ludi Qin[1], Zhenyang Zhong[1], Zhenghua An[1,3,4,5,*] Lidong Chen[6], and Wei Lu[2,7,*]

[1] State Key Laboratory of Surface Physics and Department of Physics, Institute for Nanoelectronic Devices and Quantum Computing, Fudan University, Shanghai 200433, Peoples Republic of China

[2] National Laboratory for Infrared Physics, Shanghai Institute of Technical Physics, Chinese Academy of Sciences, Shanghai 200083, China

[3] Shanghai Qi Zhi Institute, 41th Floor, AI Tower, No. 701 Yunjin Road, Xuhui District, Shanghai, 200232, China

[4] Yiwu Research Institute of Fudan University, Chengbei Road, Yiwu City, 322000 Zhejiang, China

[5] Zhangjiang Fudan International Innovation Center, Fudan University, Shanghai 201210, China

[6] State Key Laboratory of High Performance Ceramics and Superfine Microstructure, Shanghai Institute of Ceramics, Chinese Academy of Science, Shanghai, China

[7] School of Physical Science and Technology, ShanghaiTech University, Shanghai, 201210, China

[†]These authors contributed equally to this work.
[*]Correspondence and requests for materials should be addressed to Z.A. (email: anzhenghua@fudan.edu.cn) and W.L. (email: luwei@mail.sitp.ac.cn).

Contents

Supplementary Notes

Supplementary Note 1. Nano-constriction device fabrication.
Supplementary Note 2. Calculation of carrier concentration.
Supplementary Note 3. Detecting electron temperature by Scanning Noise Microscope.
Supplementary Note 4. Detecting the polarity of the T_L and T_e by SThM and SNoiM.
Supplementary Note 5. Linear Peltier effect in Chromium–Silicon heterostructure.
Supplementary Note 6. Simulation for nonequilibrium feature between electron and lattice.
Supplementary Note 7. Simulation of temperature profile due to conventional Thomson effect.
Supplementary Note 8. Extracting the nonequilibrium thermoelectric signals by simulation.
Supplementary Note 9. Measurement of local Seebeck coefficient change via active heated-probe local thermovoltage measurement.

Supplementary Figures

Fig. S1. Structure and current characteristic curve of test Nano-constriction device.
Fig. S2. The structure and results of the Hall experiment.
Fig. S3. Results of SNoiM measurements.
Fig. S4. Bipolar AC measurements of T_L and T_e.
Fig. S5. SThM experiment in Cr-Si interface.
Fig. S6. Measured 1D profiles of $\dot{Q}_{1f}$(x) and $\dot{Q}_{3f}$(x).
Fig. S7. Two temperature model for Si nano-constriction device in COMSOL.
Fig. S8. Simulated $|\mathbf{E}|$ of Si nano-constriction device at V_b=10 V.
Fig. S9. Reproducing the nonequilibrium signatures in simulations at V_b=10 V.
Fig. S10. Simulations for the conventional Thomson effect under quasi-equilibrium condition.
Fig. S11. The simulated current-polarity dependent T_L under ±10 V.
Fig. S12. Active heated-probe local thermovoltage measurement.

Supplementary References 1-28

Supplementary Note 1. Nano-constriction device fabrication

Fig. S1(a) shows the schematic structure of the Si nano-constriction device fabricated by electron beam lithography (EBL) and inductive coupled plasma-reactive ion etching (ICP-RIE). The etching depth of about 115 nm is measured by mechanical profilometry, ensuring the conducted film outside of the channel has been totally etched. A narrow constricted conductive region with 400 nm width (as shown in Fig. S1(b)) is connected to the source and drain via the aluminum (~90 nm)-silicon ohmic contacts (as demonstrated in Fig. S1(c)). The cross-shaped gold stripes (~100 nm) are served in SNoiM measurements for guiding the center of the focal spot and the narrowest position.

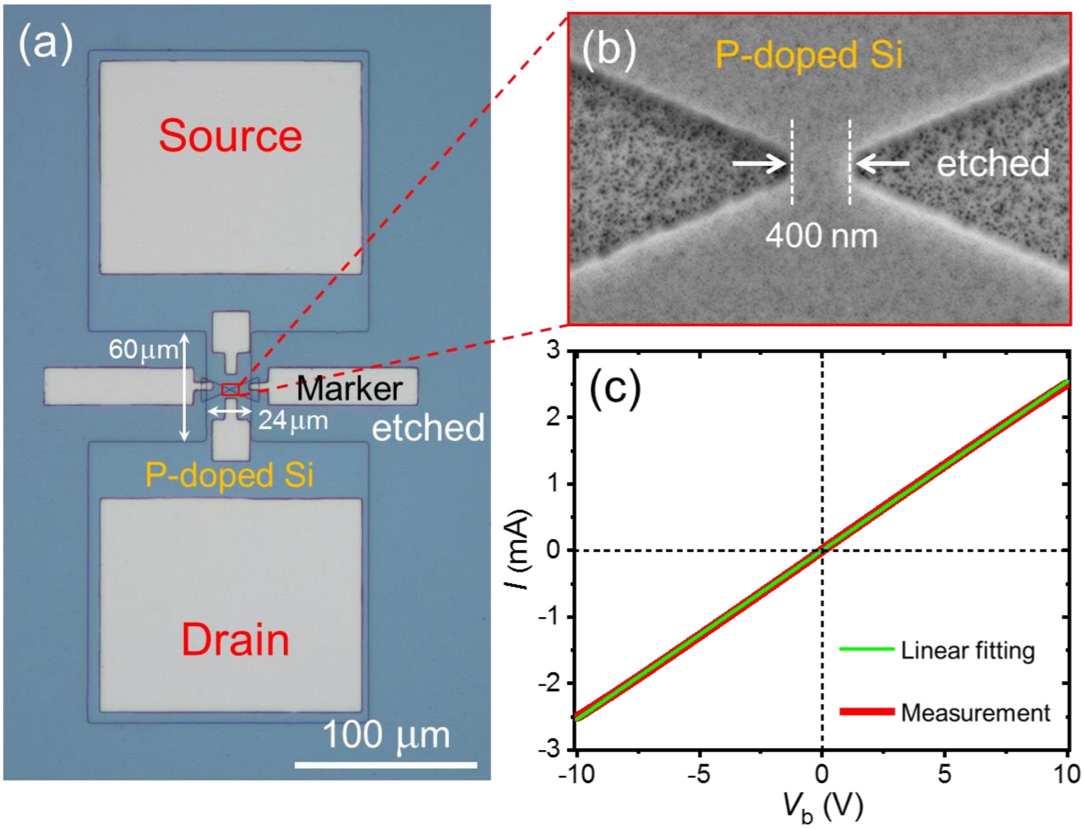

Fig. S1 Structure and current characteristic curve of test Nano-constriction device: (a) Optical micrograph for sketching the structure of the Si device and (b) SEM image of the narrowest region (~400 nm width); (c) *I*-*V* curve of nano-constriction Si device, the measured drain-source current *I* (red line) exhibits a good linear relationship with bias voltage V_b (as indicated by the green linear-fitted line), which demonstrates the good Ohmic contact between P-doped Si channel and drain/source electrodes.

Supplementary Note 2. Calculation of carrier concentration

P-doped Si film is fabricated into a Hall bar structure (as shown in Fig. S2(a)) for determining the carrier concentration and electric conductivity of the device, both the parameters are essential for the numerical simulations. The width (*W*) of the Hall bar channel is 100 μm, and the distance between the longitudinal contacts (V_R- and V_R+) is 340 μm.

The electron's concentration N_{3D} can be calculated by:

$$N_{3\mathrm{D}} = \frac{1}{d} \cdot \frac{I}{V_{\mathrm{H}}} \cdot \frac{B}{e} \tag{S1}$$

Where I, V_{H}, B, e and d are the current, Hall voltage, applied magnetic field (±300 mT), elementary electric charge and thickness of P-doped Si film (90 nm), respectively. It should be noted that, to eliminate the contribution to V_{H} from the inevitable misalignment between the pair of Hall contacts (V_{H}- and V_{H}+), the values of V_{H} here under both positive and negative magnetic field has been subtracting the background (transvers voltage under 0 T). Fig. S2(b) clearly show the linear relationship of V_{H} with I, where the data measured under both positive (+300 mT; red dots) and negative (-300 mT; blue dots) magnetic field almost coincide with each other, demonstrating the contribution from the misalignment has been well eliminated. The average value of $\left|\frac{I}{V_{\mathrm{H}}}\right|$ in S1 is acquired by linear fitting (gray line) from the measured data. Therefore, the concentration $N_{3\mathrm{D}}$ is calculated about 0.95×10^{19} cm^{-3}.

The electrical conductivity σ can be deduced from:

$$\sigma = \frac{1}{\rho} = \frac{L}{\bar{R}W}$$

Where $\bar{R}$ is the average resistance between the longitudinal contacts (V_{R}- and V_{R}+), which is obtained from the I-V_{R} data by linear fitting (as shown in Fig. S2(c)). As results, σ is calculated about 1.74×10^{4} S/m, which is consistent with the previously reported value for this concentration (P-doped)[1,2].

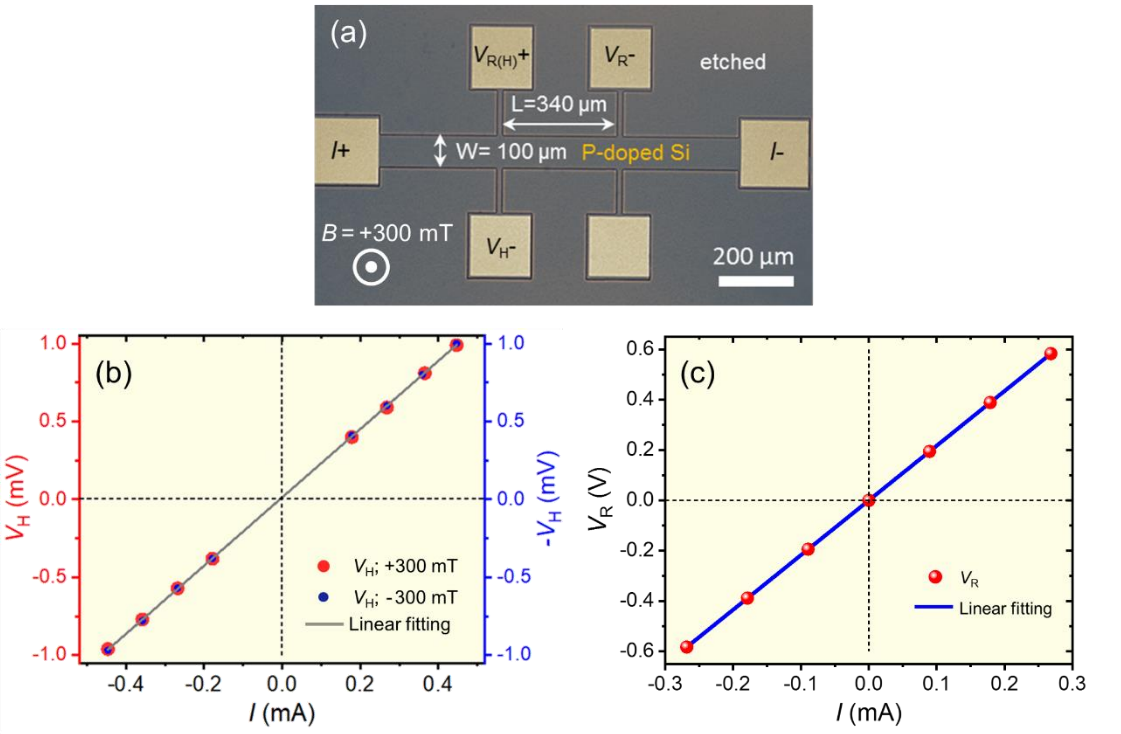

Fig. S2 The structure and results of the Hall experiment: (a) Optical image of the Hall bar device; (b) Experiment results (red and blue points) and linear fitting (grey line) of Hall test under +/-300 mT (the positive direction of the magnetic field is shown in Fig. S2(a)); (c) The measured current data (red points) under different bias voltage and the fitting curve (blue line) indicates an ideal linearity of the device.

Supplementary Note 3. Detecting electron temperature by Scanning Noise Microscope

To mapping the local temperature of the electron, a kind of scattering Scanning Nearfield Optical Microscope(s-SNOM), which can be also called Scanning Noise Microscope (SNoiM)[3-6] is applied for electron temperature measurement. Moving electrons can generate fluctuating electromagnetic (EM) evanescent field which is supposed to be related to the average of electrons' kinetic energy. This evanescent field will propagate to the far-field with a nanoscale tungsten tip approaching the surface of the sample, then collected by the confocal optical system and detected by a sensor. The nearfield signal detected by SNoiM can be written as[7,8]:

$$u(\mathrm{z},\omega) = \rho(\mathrm{z},\omega)\frac{\hbar\omega}{exp(\frac{\hbar\omega}{k_{\mathrm{B}}T})-1}$$

Where $\rho(\mathrm{z},\omega)$ is EM local density of states, z is the distance between tip and sample surface, ω is the angular frequency of the EM wave, $\hbar$is the Dirac constant and $\mathrm{k_B}$ is the Boltzmann constant. While the EM local density of states would be merely dependent by the material with invariant z and ω , the electron temperature turns out to be the only parameter producing influence to the nearfield signal $\mathrm{u(z,\omega)}$. All the spatial profiles of $\mathrm{u(z,\omega)}$ can be converted to electron temperature by comparison with signal under zero external electrical field (note that the experiment is completed under room temperature, so the electron temperature should be $T_0 = 300$ K under 0 V):

$$\frac{V}{V_0} = \frac{exp(\frac{\hbar\omega}{k_B T_0}) - 1}{exp(\frac{\hbar\omega}{k_B T}) - 1}$$

Consequently, with the nearfield signal (V_{NF}) shown in Fig. S3(a), the line profiles of electron temperature along the nano-constriction under different applied voltages in fig. 4(b) can be calculated. However, the nearfield signal under 0 V ($\mathrm{V_0}$) is extremely weak and cannot be measured by current modulation method, so the tip-height modulation is applied for extracting $\mathrm{V_0}$ and the decay curve of nearfield signal under 0 V and 10 V are shown in Fig. S3(b). There is also a peak in the narrowest area, however the temperature of the electron is higher than the lattice's results shown in Fig. 3.

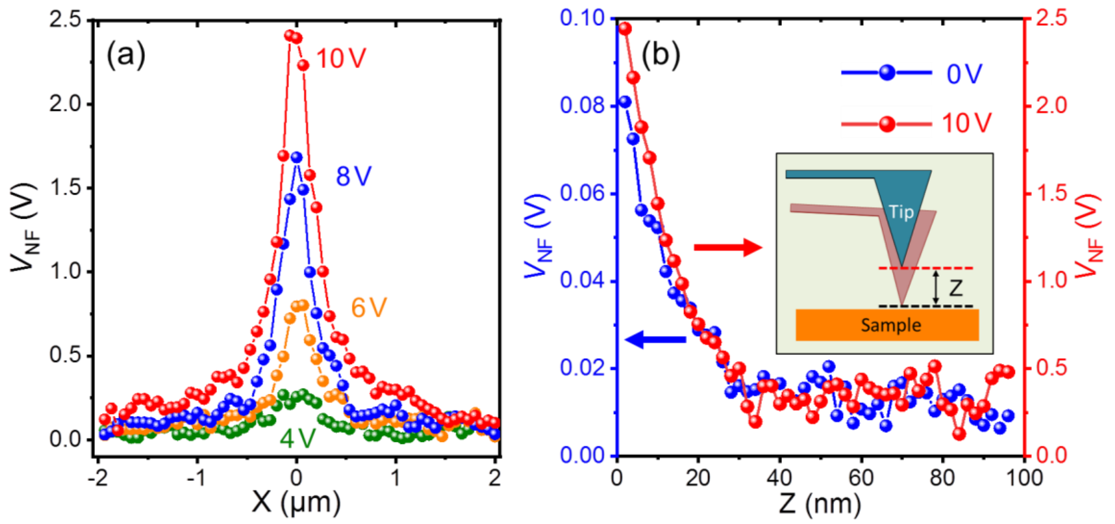

Fig. S3 Results of SNoiM measurements: (a) 1-D nearfield signal distribution along the center of nano-constriction under different applied voltage; (b) The relationship between the nearfield signal and tip height (decay curve) in the center of Nano-constriction device in Fig. S1

Supplementary Note 4. Detecting the polarity of the T_L and T_e by SThM and SNoiM.

The Scanning Thermal Microscope (SThM) is equipped with a lock-in amplifier, which allows directly observing the certain thermal signals from lattice system with high sensitivity of temperature and spatial resolution (better than 50 mK and 50 nm)[5,9], such as Joule heating, TE cooling/heating, and the total lattice temperature (T_L). In order to explore the current-polarity dependence of T_L under nonequilibrium condition, AC square-waves bias voltage of 10 V (413 Hz) with appointed polarity is supplied to the device (as illustrated in Fig. S4(a)) while the SThM scanning the thermal profile of a fixed line at the constriction (scanning distance is 5 μm). The one-dimensional T_L distributions for both directions of current obtained by the above method are shown in Fig. S4(b), in which the profiles clearly exhibit the current-polarity dependence: the profiles are shifted toward the direction of the carrier (electron; n-type silicon) traveling while the peak values are almost the same, leading to clear split between the profiles. To determine the narrowest position (the middle point) of constriction, we suppose that the deviated distance of profiles with opposite polarities are the same (but in the opposite direction), as a result, the middle position is defined by the crossover point of the profiles (x_0=2.54 μm) that marked by the black dashed line in Fig. S4(b). Consequently, the deviation of T_L relative to the middle position in Fig. 1(c) is well defined by this method. Owing to the large gradient of T_L in the vicinity of constriction, the current-polarity dependence would lead to a considerable temperature difference ($T_L^+(x) - T_L^-(x)$) between positive ($T_L^+(x)$) and negative ($T_L^-(x)$) biased conditions, which can be obtained by means of square-waves modulated voltage with ±10 V (Fig. S4(c)). As shown in Fig. S4(d), the thermal profile of $T_L^+(x) - T_L^-(x)$exhibit a strong antisymmetric feature with the opposite signs on both sides of constriction center, the

maximum value approach to 3 K indicating the appearance of the considerable nonequilibrium thermoelectric (TE) effect in T_L. In principle, the net TE component is equal to[10,11]:

$$T_{TE}^{+}(x) = \frac{T_L^{+}(x) - T_L^{-}(x)}{2}$$

Accordingly, the TE cooling/heating as shown in Fig. 2 and Fig. 3 that acquired from the original data lock-in amplifier by the above method should be additionally multiplied by $1/2$.

As a comparison, Figs. S4(e) shows the 1-D T_e profiles measured with SNoiM under unipolar pulsed bias 0/+10V (red) and 0/-10 V modulated at 5 Hz. Unlike the data in Fig. S4(b) (SThM), no peak shift can be identified in Fig. S4(e) between positive and negative bias. Consistently, the bipolar pulsed bias (+10V/-10V modulated at 5Hz) as shown in Fig. S4(f) gives no discernible signal like SThM measurement (Fig. S4(d)).

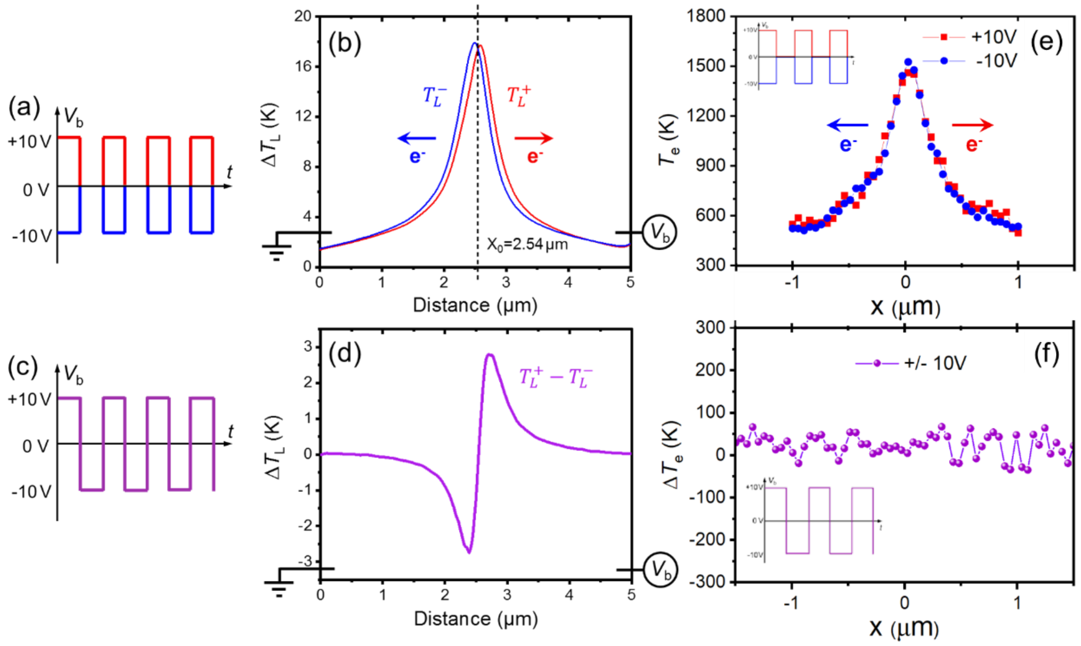

Fig. S4 Bipolar AC measurements of T_L and T_e: (a) 0~+10 V (or -10 V) square wave input to the device for detecting the total heat; (b) The 1-D SThM results of the Si nano-constriction device under bias voltage of ±10 V (413 Hz), as shown in Fig. S4(a); (c) -10 V~+10 V (413 Hz) square wave input to the device for detecting the TE signals; (d) The 1-D SThM results under bias voltage as shown in Fig. S4(c). (e) The 1-D T_e profiles under bias voltage of ±10 V (5 Hz). (f) Bipolar AC measurement of 1-D T_e profiles (-10 V~+10 V, 5 Hz).

Supplementary Note 5. Linear TE Peltier effect in Chromium–Silicon heterostructure

The conventional Peltier effect was predicted to appear at the junction composed of dissimilar materials with different Seebeck coefficients, the Peltier cooling/heating power at the interface is determined according to: $\dot{Q}_{Peltier} = \Pi_{ab} I$, where $\Pi_{ab} = S_{ab} T = (S_a - S_b)T$ is the differential Peltier coefficient of the junction, S_a and S_b

denote the absolute Seebeck coefficient of the materials, hence

$$\dot{Q}_{\text{Peltier}} = (S_{\text{a}} - S_{\text{b}})TI \quad \text{(S2)}$$

In most cases of conventional thermoelectric devices, the temperature variation can be neglected, correspondingly the temperature T in equation S2 can be treated as a constant, hence the Peltier signals depend linearly on the current. Despite the Peltier effects have been experimentally observed in the various types of the junction, such as metal-graphene[12,13], metal-organic film[11], and metal-semiconductor[14,15], the quantitative analysis for linear dependence on the current of Peltier effect is still lacking. More importantly, to emphasize the reliability of Peltier signal detection, and eliminate the possible artefacts that may lead to a third-power dependence of current in the nonequilibrium thermoelectric data, a conventical thermoelectric device with a metal-semiconductor junction structure is fabricated. As sketched in Fig. S5(a), the device is composed of a P-doped silicon (n-type: ~$1\times10^{19}/cm^3$) segment with two Chromium (Cr) electrodes which are fabricated on silicon substrate (high-resistivity). The Peltier signals here are detected by using the AC square-waves bias voltage (as described in S4) with 213 Hz, and the Joule heating are measured by the sinusoidal-wave-modulated voltage (213 Hz), details for measuring method are described in the method. As shown in Fig. S5(d), when the bias voltage is supplied to the device, the Peltier cooling/heating effect appears at the junction as the current flow through the Cr/Si interfaces, owing to the dissimilar Seebeck coefficient of the P-doped (~$1\times10^{19}/cm^3$) Si (about hundreds of -μV/K)[2,16,17] and Cr (~20 μV/K)[18] according to the equation S2, where the sign of the Peltier signals are determined by the current direction and the sign of the differential Seebeck coefficient (S_{SiCr}). It should be noted that the Peltier signals are slightly concentrated toward the edges of Cr contacts, such phenomenon has also been observed in the metal-graphene heterojunction which is attributed to the current crowding induced non-uniform current density over a finite length in metal contacts[12] (as indicated in Fig. S5(a) right by red arrows). Whereas the Joule heating is concentrated around the center of the silicon segment (Fig. S5(c)) due to the relatively large resistivity of the doped silicon and the more concentrated current density (Joule-Lenz law). Fig. S5(e) and (f) show the 1D thermal profiles of Joule heating and Peltier cooling/heating under various bias voltages of 2-4 V that are measured along the junction, which allowed for quantitative analysis the signals. The peak values of Joule heating extracted from 1D profiles under various voltages are shown in Fig. S5(g), clearly showing quadratic dependence of the current (replace the voltage owing to the linear relationship between the bias voltage and current, as indicated in Fig. S5(b)) by the fitted line. In contrast to the nonequilibrium TE effect, the Peltier signals (absolute maximum values of Peltier cooling) here exhibit the linear relation with current (as indicated by linear fitting), consistent with the theoretical prediction from equation S2.

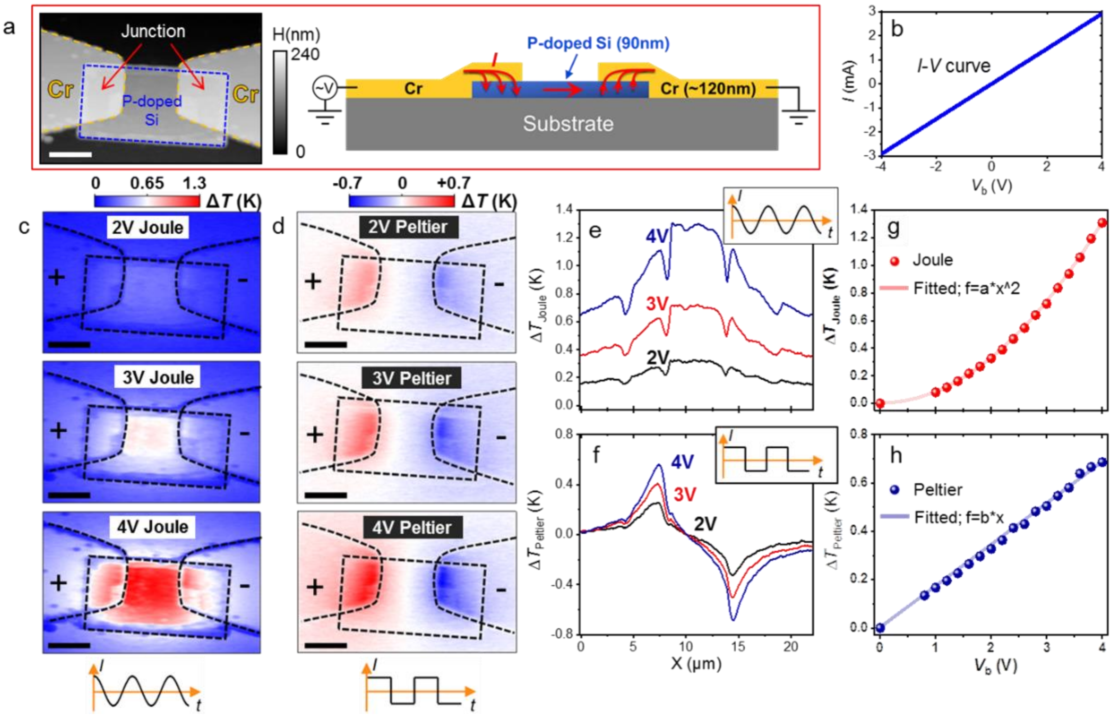

Fig. S5 SThM experiment in Cr-Si interface: (a) Schematic structure (right) and AFM topography (left) of the Cr-Si interface device; (b) *I-V* curve of the Cr-Si interface device; 2D image of (c) Joule heating and (d) Peltier heating under different bias voltage; The line profile of (e) Joule heating and (f) Peltier heating under different applied voltage; The measured data and fitting curve of (g) Joule heating and (h) Peltier heating in the center under various applied voltage.

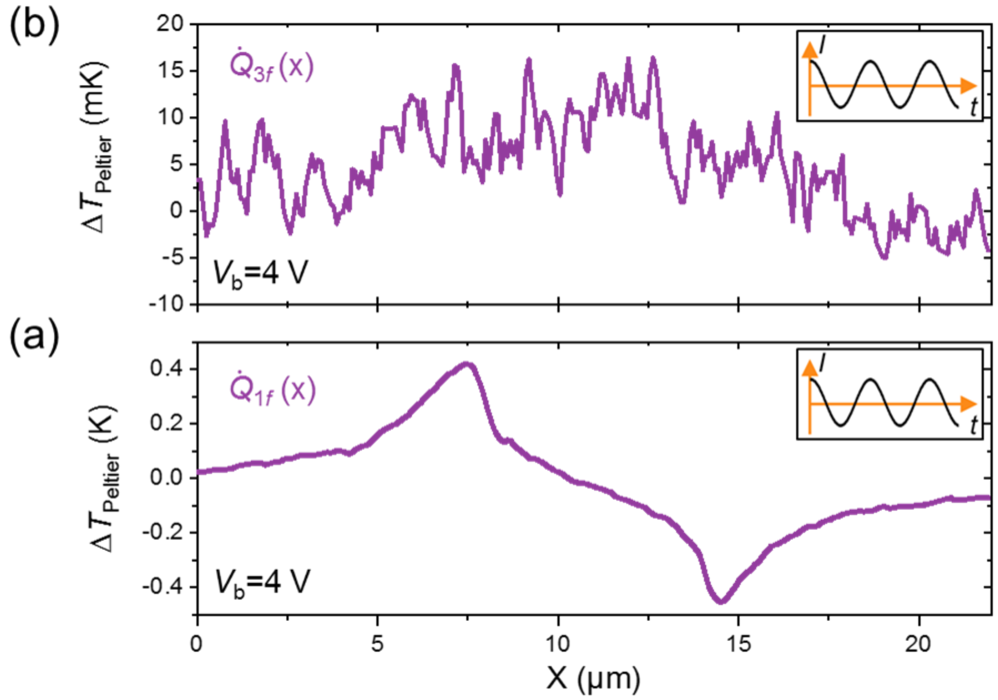

Fig. S6 Measured 1D profiles of $\dot{Q}_{1f}(x)$ (a) and $\dot{Q}_{3f}(x)$ (b) along the channel of Cr-Si device at V_b=4 V, which are demodulated at the first and third harmonic by the lock-in

technique.

Supplementary Note 6. Simulation for nonequilibrium feature between electron and lattice

To explore the nonequilibrium signature between electron and lattice in nano-construction of Silicon under high electrical field, we adopted a recently developed simulation method[19] based on the two-temperature model for extracting the distributions of T_e and T_L from the respective subsystems. The simulations are performed by commercial Multiphysics software (COMSOL). Considering the practical computing time, the modeled geometry is simplified by a constriction structure (Fig. S7(a)) with the narrowest width of 400 nm (Fig. S7(b)), and the thickness of the substrate is down to 5 μm for the same reason. To ensure that the simulated distributions of electric field and current density around the narrowest region are in accord with those in the real device under the same bias conditions, we choose the current source instead of the voltage source for simulations, where the currents under various bias voltages are deduced from the *I-V* curve (see Fig. S1(c).

In this model, the conducting layer (P-doped Silicon film) is physically separated into electron- and the lattice layers, as shown in Fig. S7(c). The electron layer represents the electron subsystem, which can be characterized by the free electron density $N_{3\mathrm{D}}$, the electrical conductivity σ_{e}, the electron thermal conductivity κ_{e}, and the electronic specific heat C_e. $N_{3\mathrm{D}} \sim 1 \times 10^{19}\ \mathrm{cm}^{-3}$ and $\sigma_{\mathrm{e}} = 1.74 \times 10^{4}\ \mathrm{S/m}$ are directly measured by the Hall device (as described in section S2), while κ_{e} and C_{e} are given by the formulas[19,20]:

$$\kappa_{\mathrm{e}} = \frac{\pi^2 N_{3\mathrm{D}} k_{\mathrm{B}}^2 T}{3m^*} \tau_{\mathrm{F}} \tag{S3}$$

and

$$C_{\mathrm{e}} = (3/2) N_{3\mathrm{D}} k_{\mathrm{B}} \tag{S4}$$

respectively, where k_{B}, m^* and τ_{F} are the Boltzmann's constant, the effective mass of conductive electron and the energy relaxation time of the free electrons. m^* of conduction band with ellipsoidal constant energy surfaces is calculated by sum over the effective masses in the different minima along the equivalent directions: $m^* = \frac{3}{(1/m_l + 1/m_t + 1/m_t)} = 0.26 m_0$ ($m_l = 0.89 m_0$ and $m_t = 0.19 m_0$ denote the longitudinal and the transverse effective mass of silicon, m_0 being the free electron mass)[21]. As for τ_{F}, there is no accurate value available, only an approximate range with the upper limit of about 120 fs[22-25] would be used for the estimation. Here, to match the experimental results, τ_{F} is taken 60 fs (half of the upper limit) in simulations. As results, κ_{e} is calculated to be 0.48 W/mK by equation (S3), which is comparable to the reported data [supplementary information of ref. 26]. The lattice subsystem of the conducting layer is characterized by the thermal conductivity[27] $\kappa_{\mathrm{L}} = 40\ \mathrm{W/mK}$ and the specific heat $C_{\mathrm{L}} = 1.63 \times 10^{6}\ \mathrm{J/K \cdot m^3}$.

Figure. S7(d) sketches the diagram of energy/heat flow in the model. Initially, the conduction electrons are excited into a nonequilibrium state by the high electric field $\mathbf{E}(\mathbf{r})$ through $P = \mathbf{J}(\mathbf{r}) \cdot \mathbf{E}(\mathbf{r}) = \sigma_{\mathrm{e}} E^2$, and consequently, the excess energy of the hot

electron would be diffusive to the surrounding electrons by electron heat conduction ($-\kappa_{\mathrm{e}}\nabla T_{\mathrm{e}}(\mathrm{x,y})$) or released to lattice via electron-phonon interaction: $(T_{\mathrm{e}}(\mathrm{x,y}) - T_{\mathrm{L}}(\mathrm{x,y}))/h_{\mathrm{e-ph}}$, where $h_{\mathrm{e-ph}} = \tau_{\mathrm{e-ph}}/C_{\mathrm{e}}^{*}$ is the interface thermal resistance between electron and lattice layer ($\tau_{\mathrm{e-ph}} = 0.26$ ps[25,28] is the electron-phonon energy relaxation time and $C_{\mathrm{e}}^{*} = (3/2)N_{\mathrm{2D}}k_{\mathrm{B}}$ is the electronic specific heat per unit area). After released to the lattice subsystem, the heat transferred within the lattice by $-\kappa_{\mathrm{L}}\nabla T_{\mathrm{L}}(\mathrm{x,y})$ or spreads into the substrate via the thermal resistance h_{I} (we assume 0 $\mathrm{Km^2/W}$ for h_{I}, owing the homoepitaxy of Si film) between lattice and substrate, and eventually spread to the heat sink (300 K) through the thermal conductivity of substrate κ_{Sub}. Parameters used in the model are summarized in Table S1.

Figure. S8 shows the simulation of the electric field strength $|\mathbf{E}|$ at $V_{\mathrm{b}} = 10$ V ($I = 2.51$ mA). The 2D distribution (Fig. S9(a)) of $|\mathbf{E}|$ is intensively concentrated at the narrowest region with the maximal value approach of 25 kV/cm with a sharp 1D profiles (Fig. S8(b)) along the channel, as indicated by the red dashed line in Fig. S8(a).

The nonequilibrium signatures between electrons and lattice at V_{b}=10 V are reproduced in the simulation results. As shown in Fig. S9, the distributions of T_{e} (Fig. S9(a)) and T_{L} (Fig. S9(b)) extracted from the respective layers exhibit the similar features with the experiments (as shown in Fig. 1) where the peak values of T_{e} (~ 1600 K) and T_{L} (~340 K) are well matched with the experimental results (~1500 K and ~320 K). Moreover, the contour of the hot spot for T_{L} is broader than that for T_{e}, such a feature is clearly elucidated by plotting 1D profiles of T_{e} and T_{L} together in Fig. S9(c), reproducing the similar line feature in Fig. 1(c). The dissimilarity in line shapes between T_{e} and T_{L} is attributed to the differences in specific heat and thermal conduction between electron and lattice by orders of magnitude (see Table S1).

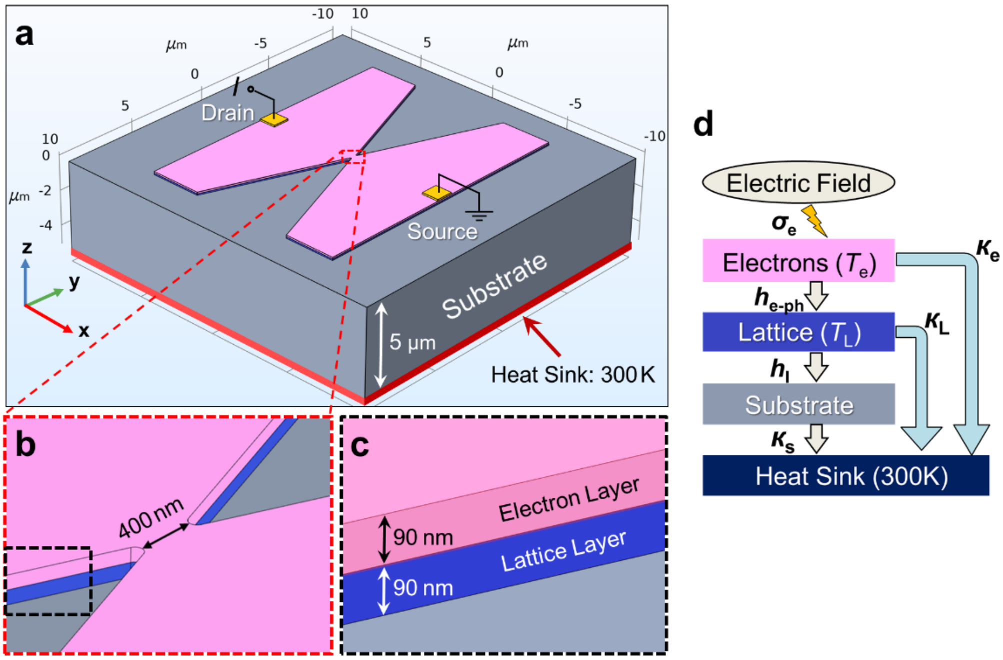

Fig. S7 Two temperature model for Si nano-constriction device in COMSOL: (a)

Schematic diagram of two temperature model; (b) Simulated structure of Si device and (c) (d) its details.

Table S1: Parameters used for the two-temperature model.

Quantity	σ_e	κ_e	C_e	κ_L	$C_{L(sub)}$	$h_{e\text{-}ph}$	h_I	κ_{Sub}
Unit	S/m	$W/(m{\cdot}K)$	$J/(m^3{\cdot}K)$	$W/(m{\cdot}K)$	$J/(m^3{\cdot}K)$	Km^2/W	Km^2/W	$W/(m{\cdot}K)$
Value	1.74×10^4	0.48	207	40	1.63×10^6	2.1×10^{-9}	0	148

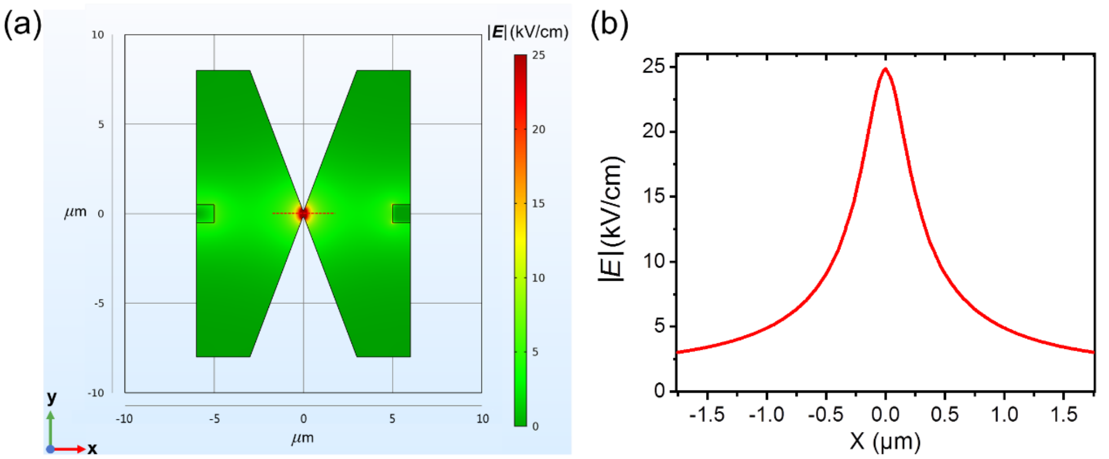

Fig. S8 Simulated |**E**| of Si nano-constriction device at V_b=10 V: (a) The 2D distribution of |**E**|; (b) The line profile of the |**E**| along the red dashed line in Fig. S9 (a).

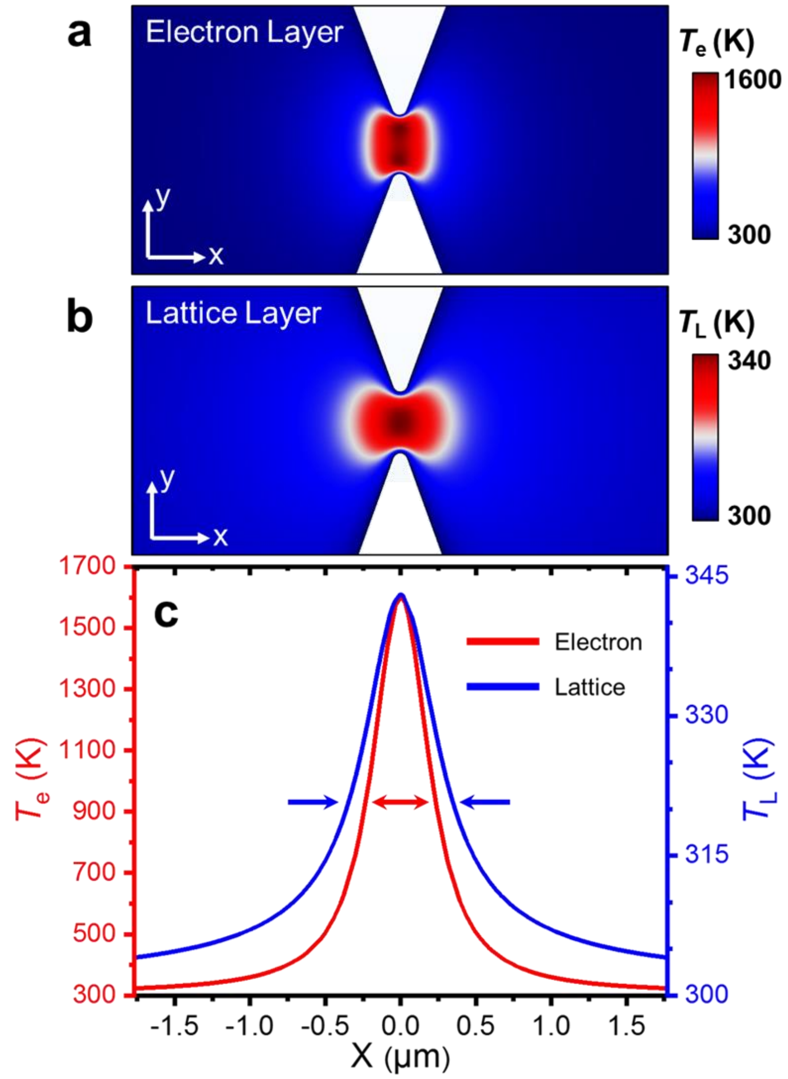

Fig. S9 Reproducing the nonequilibrium signatures in simulations at V_b=10 V: (a) electron temperature and (b) lattice temperature distributions in the simulation results; (c) comparison of 1D thermal profiles of electron and lattice temperature.

Supplementary Note 7. Simulation of temperature profile due to conventional Thomson effect.

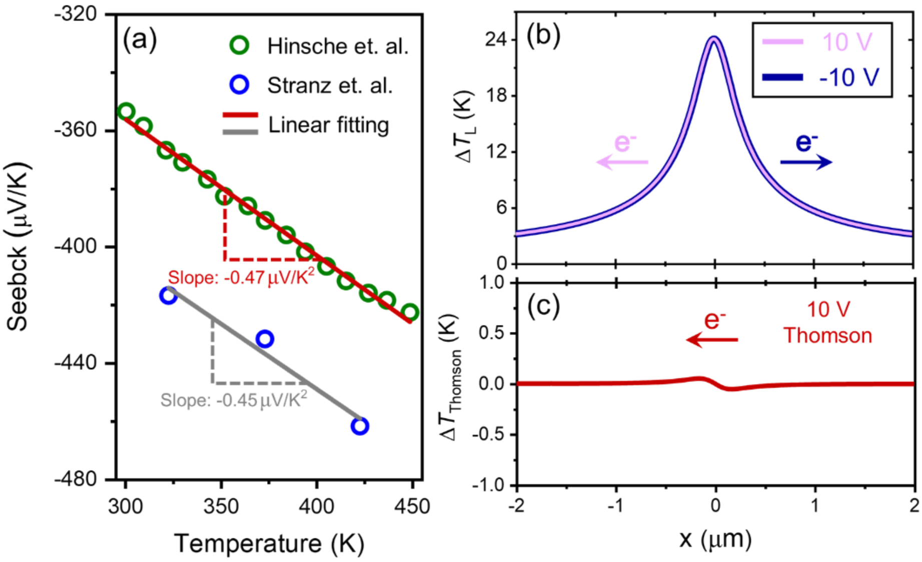

Fig.S10 Simulations for the conventional Thomson effect under quasi-equilibrium condition: (a) Reasonable local Seebeck coefficient change due to temperature rise at the nanon-constriction (Data taken from Hinsche et al and Stranz et al (Ref. 48)). (b) Simulated lattice temperature profiles for +10 V and -10 V bias voltages. The deviation is dramatically smaller than our experimental data (Figs. 2d-h, Fig.3b). (c) Derived Thomson signal profile (V_b=10 V) with the amplitude of the asymmetric curve being only roughly ~4 % of the real observed signal under the same bias (Fig. 3b).

Supplementary Note 8. Extracting the nonequilibrium thermoelectric signals by simulation

The simulations for the nonequilibrium Thermoelectric effects are implemented by a strategy that merges the electron and lattice layer as a whole uniform conducting layer (90-nm thick) where the lateral dimensions are consistent with that of the two-temperature model (as shown in Fig. S9(a)). This model includes heat transfer in solid, electrical current and thermoelectric effects. The main equations solved for this system is:

$$\begin{cases} \rho C \dfrac{dT}{dt} = \nabla \cdot (\kappa \nabla T) - \nabla \cdot (\Pi \mathbf{J}) + Q \\ Q = \mathbf{J} \cdot \mathbf{E} \\ \mathbf{J} = \sigma \mathbf{E};\ \nabla \cdot \mathbf{J} = 0 \end{cases}$$

Where ρ, C, σ and κ are the material properties of density, heat capacity, and electrical/thermal conductivities, respectively. T, Q, Π, $\mathbf{J}$ and $\mathbf{E}$ are the temperature, heat density, Peltier coefficient, current density, and electric field.

The above equation applies for the quasi-equilibrium case where distinguishing T_e and T_L become unnecessary. In present work, SThM and SNoiM results show

unambiguously the occurrence of nonequilibrium condition, i.e., $T_{\mathrm{e}} \gg T_{\mathrm{L}}$. As a consequence, the thermal conductance term ($\nabla \cdot (\kappa \nabla T)$) in above equation should be renewed to include both electron and lattice contributions; the thermoelectric term ($-\nabla \cdot (\Pi \mathbf{J})$) should be replaced with nonequilibrium thermoelectric coefficient using electron temperature T_{e} because thermoelectric transport is realized by transport of hot electrons with effective temperature (T_{e}). Considering the steady-state which SNoiM mainly probes, the above equation can be transformed into,

$$\frac{j^2}{\sigma} + \sum_{\mathrm{i=e,L}} \nabla \cdot (\kappa_{\mathrm{i}} \nabla T_{\mathrm{i}}) - \nabla (S T_{\mathrm{e}}) \cdot \mathbf{J} = 0$$

Where S is the effective Seebeck coefficient and $\Pi_{\mathrm{Nonequilibrium}} = S T_{\mathrm{e}}$ is the nonequilibrium thermoelectric coefficient. This equation is same as Eq. (1) in the main text. From this equation, the measured $\Delta T_{\mathrm{Peltier}}^{+/-}$ in SThM experiments is the term of $-\nabla (S T_{\mathrm{e}}) \cdot \mathbf{J}$. It can be further decomposed into $-S \nabla T_{\mathrm{e}} \cdot \mathbf{J}$ and $-T_{\mathrm{e}} \nabla S \cdot \mathbf{J}$. It turns out that the first term ($-S \nabla T_{\mathrm{e}} \cdot \mathbf{J}$) dominates in our non-equilibrium Si device due to large gradient of T_{e}, and the second term ($-T_{\mathrm{e}} \nabla S \cdot \mathbf{J}$) contributes only a very small fraction (see Supplementary Note 7). The dominant term is apparently different from conventional Peltier or Thomson effects ($\dot{Q}_{\mathrm{Conventional\ Peltier}} = (\Pi_{\mathrm{A}} - \Pi_{\mathrm{B}}) \mathbf{J}$ and $\dot{Q}_{\mathrm{conventional\ Thomson}} = -\beta \nabla T_{\mathrm{L}} \cdot \mathbf{J} = -\left(T_{\mathrm{L}} \frac{dS}{dT_{\mathrm{L}}}\right) \nabla T_{\mathrm{L}} \cdot \mathbf{J}$).

The nonuniform conductor structure in our work (i.e., nanoconstriction) makes most physical quantities nonuniform including T_{e} and hence $\dot{Q}_{\mathrm{Conventional\ Peltier}}$. The distribution of the Peltier coefficient under nonequilibrium condition is governed by local electron temperature: $\Pi(\mathrm{x,y}) \approx \frac{\pi^2}{3e} k_{\mathrm{B}} T_{\mathrm{e}}(\mathrm{x,y})$, which can be deduced by the two-temperature model at the corresponding bias. In this model, the variations of the lattice temperature ΔT_{L} include both the symmetric Joule heating ($\Delta T_{\mathrm{Joule}}$) and the antisymmetric TE cooling/heating (ΔT_{TE}):

$$\Delta T_{\mathrm{L}}^{+/-} = \Delta T_{\mathrm{Joule}} + \Delta T_{\mathrm{TE}}^{+/-}$$

Here, the sign (+/-) of ΔT_{L} and ΔT_{TE} denote the current polarities, where $\Delta T_{\mathrm{TE}}^{+} = -\Delta T_{\mathrm{TE}}^{-}$. As shown in Fig. S11, the lattice temperature $T_{\mathrm{L}}^{+/-} = \Delta T_{\mathrm{L}}^{+/-} + 300$ K with opposite current-polarity (current source of ±2.51 mA substitute for V_{b}=±10 V) exhibit the asymmetric features owing to the component of the TE cooling/heating effect (Fig. S11), which are in agreement with the experimental data (as shown in Fig. S4(b)). Hence, the distributions of $\Delta T_{\mathrm{Joule}}$ and ΔT_{TE} can be separately extracted by:

$$\Delta T_{\mathrm{Joule}}(\mathrm{x,y}) = \frac{\Delta T_{\mathrm{L}}^{+}(\mathrm{x,y}) + \Delta T_{\mathrm{L}}^{-}(\mathrm{x,y})}{2}$$

$$\Delta T_{\mathrm{TE}}^{\pm}(\mathrm{x,y}) = \frac{\Delta T_{\mathrm{L}}^{\pm}(\mathrm{x,y}) - \Delta T_{\mathrm{L}}^{\mp}(\mathrm{x,y})}{2}$$

as shown in Fig. 3(d)-(f). Inaddition, it should noted that the simulated peak values of

lattice temperature (~325 K, V_b=±10 V) by this model is comparable to that in the two-temperature model (~343 K, V_b=10 V), suggesting the reliability of these models.

Table S2: Parameters in the thermoelectric model.

Quantity	σ	κ	$\rho_{(sub)}$	$C_{(sub)}$	h_I	κ_{Sub}
Unit	S/m	$W/(m{\cdot}K)$	kg/m^3	$J/(kg{\cdot}K)$	Km^2/W	$W/(m{\cdot}K)$
Value	1.74×10^4	40	2329	700	0	148

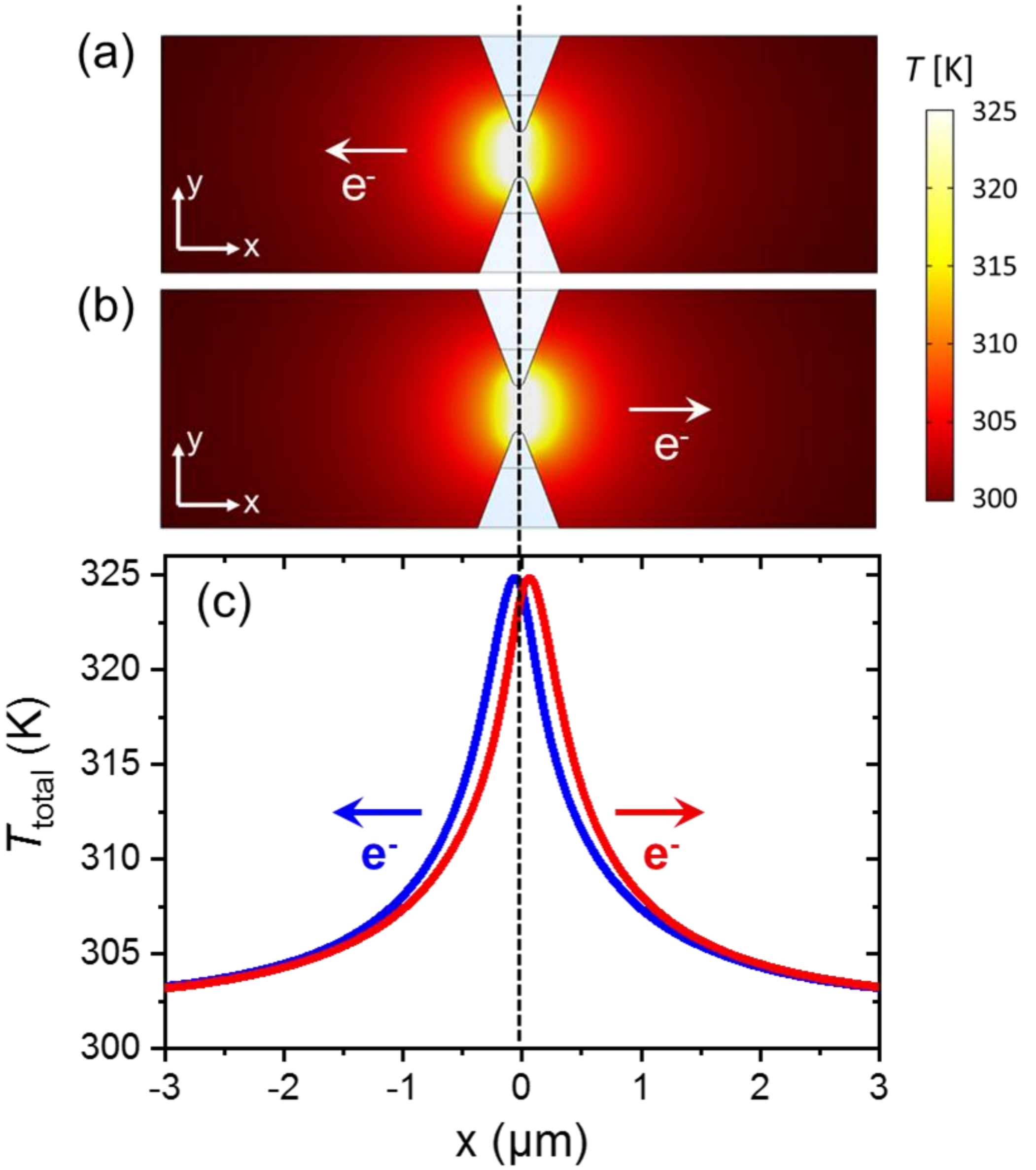

Fig. S11 The simulated current-polarity dependent T_L under ±10 V: (a) and (b) 2D distributions of $T_L(x, y)$ with opposite current polarity (±2.51 mA); (c) 1D thermal profiles of T_L that extracted from (a) and (b) along the channel in the distance of 6 μm.

Supplementary Note 9. Measurement of local Seebeck coefficient change via heated tip

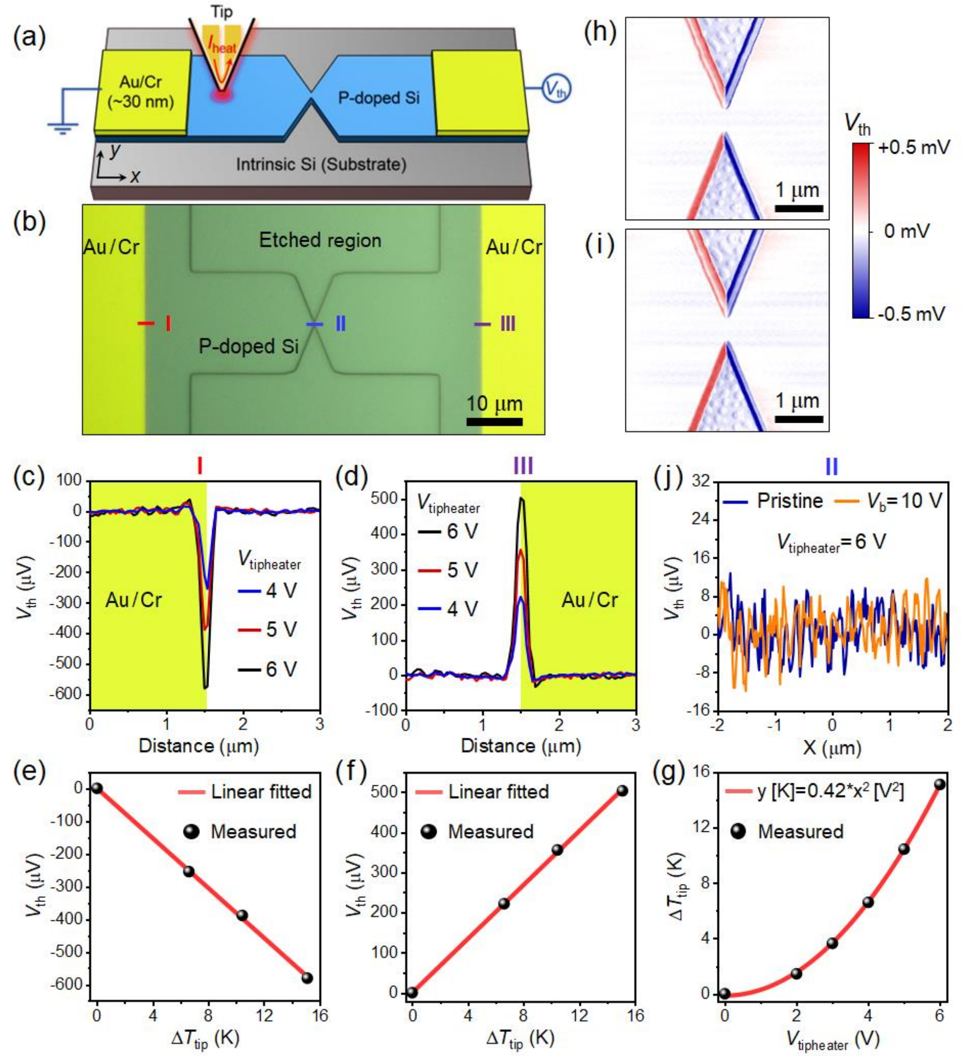

Fig. S12 Active heated-probe local thermovoltage (V_{th}) measurement: (a) Schematic measurement setup of scanning thermal gate microscopy with heated tip and (b) microscopic image of sample with I, II, III labelling the different regions. The line profiles of measured sample V_{th} at different tip heating bias ($V_{tipheater}$= 4 V, 5 V and 6 V) in region I (c) and region III (d) crossing the edge of metal electrodes. The sample V_{th} signal is proportional to the tip temperature (ΔT_{tip}) rise (i.e. temperature gradient) with negative polarity in region I (e) and positive in region III (f) according to equation S5. The temperature rise of the tip is calibrated and found to be proportional to the square of tip bias as shown in (g). In the nano-constricted region II, no apparent sample V_{th} can be obtained for both without (h) and with +10 V (i) source-drain bias training and the absence of the sample V_{th} signal is also seen in the line profiles (j). Artificial signal appears at mesa edges due to symmetry-breaking in hot-tip-induced electron diffusion (red and blue in (h) and (i)).

To measure the local Seebeck coefficient distribution, an active heated-probe local thermovoltage measurement is carried out via the heated SThM tip (NP-SThM-02, TSPNANO). The probe is heated by an AC drive (5 MHz sine wave in our experiment) and temperature of tip apex is obtained by reading the DC thermovoltage ($V_{\mathrm{tip,th}}$) signal of the probe. When the heated tip contact with the sample surface, the local temperature gradient ∇T induced by the tip would lead to the thermovoltage according to:

$$V_{\mathrm{th}} = -\int_{\mathrm{left}}^{\mathrm{right}} S(x) \cdot \nabla T(x)\, \mathrm{d}x. \tag{S5}$$

We scan the hot probe along the metal/P-doped Si boundaries and the constriction of P-doped Si channel, recording the thermovoltage of sample (according to Eq. S5). Change of Seebeck coefficient will cause thermovoltage under the temperature gradient. At the two boundaries of electrode and sample, opposite voltage is observed for positive and negative sides, as expected. Besides, by changing the temperature of the tip, liner response of thermovoltage with the temperature of the tip is shown (Figs. S12 (e), (f)), confirming the functionality of the measurements. However, no clear voltage signal has been observed at the nano-construction region for both before (Fig. S12(h)) and after (Fig. S12(i)) driving the device with 10 V bias. We therefore conclude that there is no apparent change of Seebeck coefficient at the nanoconstruction.

Supplementary References

1. Mousty, F., Ostoja, P. & Passari, L. Relationship between resistivity and phosphorus concentration in silicon. *J. Appl. Phys.* **45**, 4576-4580 (1974).
2. Bennett, N. S. Thermoelectric performance in n-type bulk silicon: The influence of dopant concentration and dopant species. *Phys. Status Solidi A* **214**, 1700307 (2017).
3. Weng, Q. *et al.* Imaging of nonlocal hot-electron energy dissipation via shot noise. *Science* **360**, 775-778 (2018).
4. Komiyama, S. Perspective: Nanoscopy of charge kinetics via terahertz fluctuation. *J. Appl. Phys.* **125**, 010901 (2019).
5. Weng, Q. *et al.* Quasiadiabatic electron transport in room temperature nanoelectronic devices induced by hot-phonon bottleneck. *Nat. Commun.* **12**, 4752-4752 (2021).
6. Yang, L. *et al.* Anisotropic Hot-Electron Kinetics Revealed by Terahertz Fluctuation. *ACS Photonics* **8**, 2674-2682 (2021).
7. Joulain, K., Mulet, J.-P., Marquier, F., Carminati, R. & Greffet, J.-J. Surface electromagnetic waves thermally excited: Radiative heat transfer, coherence properties and Casimir forces revisited in the near field. *Surf. Sci. Rep.* **57**, 59-112 (2005).
8. Biehs, S. A., Reddig, D. & Holthaus, M. Thermal radiation and near-field energy density of thin metallic films. *Eur. Phys. J. B* **55**, 237-251 (2007).
9. Shi, L. & Majumdar, A. Thermal Transport Mechanisms at Nanoscale Point Contacts. *J. Heat Transfer* **124**, 329-337 (2001).
10. Harzheim, A. *et al.* Geometrically Enhanced Thermoelectric Effects in Graphene Nanoconstrictions. *Nano Lett.* **18**, 7719-7725 (2018).
11. Jin, W. *et al.* Exploring Peltier effect in organic thermoelectric films. *Nat. Commun.* **9**, 3586-3586 (2018).
12. Grosse, K. L., Bae, M.-H., Lian, F., Pop, E. & King, W. P. Nanoscale Joule heating, Peltier cooling and current crowding at graphene-metal contacts. *Nat. Nanotechnol.* **6**, 287-290 (2011).
13. Vera-Marun, I. J., van den Berg, J. J., Dejene, F. K. & van Wees, B. J. Direct electronic measurement of Peltier cooling and heating in graphene. *Nat. Commun.* **7**, 11525-11525 (2016).
14. Könemann, F., Chen, I. J., Lehmann, S., Thelander, C. & Gotsmann, B. Imaging the Thermalization of Hot Carriers after Thermionic Emission over a Polytype Barrier. *Phys. Rev. Appl.* **13** (2020).
15. Gächter, N. *et al.* Spatially resolved thermoelectric effects in: Operando semiconductor-metal nanowire heterostructures. *Nanoscale* **12**, 20590-20597 (2020).
16. Salleh, F., Asai, K., Ishida, A. & Ikeda, H. Seebeck Coefficient of Ultrathin Silicon-on-Insulator Layers. *Appl. Phys. Express* **2**, 071203 (2009).
17. Stranz, A., Kähler, J., Waag, A. & Peiner, E. Thermoelectric Properties of High-Doped Silicon from Room Temperature to 900 K. *Journal of Electronic Materials* **42**, 2381-2387 (2013).
18. Moore, J. P., Williams, R. K. & Graves, R. S. Thermal conductivity, electrical resistivity, and Seebeck coefficient of high-purity chromium from 280 to 1000 K. *J. Appl. Phys.* **48**, 610-617 (1977).

19. Yang, L., Qian, R. J., An, Z. H., Komiyama, S. & Lu, W. Simulation of temperature profile for the electron and the lattice systems in laterally structured layered conductors. *Europhys. Lett.* **128**, 17001 (2019).
20. Hopkins, P. E. *et al.* Ultrafast thermoelectric properties of gold under conditions of strong electron-phonon nonequilibrium. *J. Appl. Phys.* **108**, 104907 (2010).
21. Nashima, S., Morikawa, O., Takata, K. & Hangyo, M. Measurement of optical properties of highly doped silicon by terahertz time domain reflection spectroscopy. *Appl. Phys. Lett.* **79**, 3923-3925 (2001).
22. Goldman, J. R. & Prybyla, J. A. Ultrafast dynamics of laser-excited electron distributions in silicon. *Phys. Rev. Lett.* **72**, 1364-1367 (1994).
23. Buhleier, R., Lüpke, G., Marowsky, G., Gogolak, Z. & Kuhl, J. Anisotropic interference of degenerate four-wave mixing in crystalline silicon. *Phys. Rev. B* **50**, 2425-2431 (1994).
24. Sjodin, T., Li, C.-M., Petek, H. & Dai, H.-L. Ultrafast transient grating scattering studies of carrier dynamics at a silicon surface. *Chem. Phys.* **251**, 205-213 (2000).
25. Sabbah, A. J. & Riffe, D. M. Femtosecond pump-probe reflectivity study of silicon carrier dynamics. *Phys. Rev. B* **66**, 165217 (2002).
26. Zhou, J. *et al.* Direct observation of large electron–phonon interaction effect on phonon heat transport. *Nat. Commun.* **11**, 6040 (2020).
27. Scott, E. A. *et al.* Simultaneous thickness and thermal conductivity measurements of thinned silicon from 100 nm to 17 μm. *Appl. Phys. Lett.* **118**, 202108 (2021).
28. Khara, G. S., Murphy, S. T., Daraszewicz, S. L. & Duffy, D. M. The influence of the electronic specific heat on swift heavy ion irradiation simulations of silicon. *J. Phys.: Condens. Matter* **28**, 395201 (2016).
